# Benefit of early treatment in inflammatory polyarthritis patients with anti–cyclic citrullinated peptide antibodies versus those without antibodies

**DOI:** 10.1002/acr.20207

**Published:** 2010-05

**Authors:** Tracey M Farragher, Mark Lunt, Darren Plant, Diane K Bunn, Anne Barton, Deborah P M Symmons

**Affiliations:** 1 Arthritis Research UK Epidemiology Unit, University of ManchesterManchester, UK; 1 Norfolk Arthritis Register, Norfolk, and Norwich University HospitalNorwich, UK

## Abstract

**Objective:**

To compare the clinical utility of anti–cyclic citrullinated peptide (anti-CCP) antibodies and rheumatoid factor (RF) testing in predicting both functional outcome and response to treatment in early inflammatory polyarthritis (IP) patients.

**Methods:**

A total of 916 IP subjects from a primary care incidence registry (1990–1994) had anti-CCP antibody and RF status determined at baseline. Mean change in Health Assessment Questionnaire (HAQ) score between baseline and 5 years was compared by antibody status. The effect of treatment with disease-modifying antirheumatic drugs and/or steroids over 5 years, early (<6 months of symptom onset) versus late initiation, and duration of treatment were also compared by anti-CCP antibody status. The analysis was adjusted for treatment decisions and censoring over the followup, using marginal structural models.

**Results:**

Anti-CCP antibody–positive patients (n = 268) had more severe disease both at presentation and 5 years of followup, and this was independent of RF. On adjustment, anti-CCP antibody–negative patients treated early experienced a significant improvement in functional disability compared with anti-CCP antibody–negative patients who were never treated (−0.31; 95% confidence interval [95% CI] −0.53, −0.08), and experienced additional benefit for each additional month of early treatment. Anti-CCP antibody–positive patients treated early did not have a significant improvement in HAQ score compared with those not treated (−0.14; 95% CI −0.52, 0.24).

**Conclusion:**

In this first observational study to examine the influence of anti-CCP antibody status on treatment response, anti-CCP antibody–positive IP patients showed less benefit from treatment, particularly early treatment, than anti-CCP antibody–negative patients. This provides support for the inclusion of anti-CCP antibodies as well as RF in the classification criteria for rheumatoid arthritis and for stratification by anti-CCP antibody status in clinical trials.

## INTRODUCTION

In the past few years, the potential role of anti–cyclic citrullinated peptide (anti-CCP) antibodies has gained increasing attention with respect to the diagnosis and classification of patients with inflammatory arthritis (1–3). In recent systematic reviews and meta-analyses, anti-CCP antibody positivity has been as sensitive as but more specific than rheumatoid factor (RF) for distinguishing rheumatoid arthritis (RA) from other forms of inflammatory arthritis (1–3). Although there is an association between the presence of RF and anti-CCP antibodies and the subsequent development of RA (4,5), anti-CCP antibodies may be detectable many years before RF and before the onset of symptoms (6). Furthermore, anti-CCP antibody positivity is associated with the development of erosions and radiologic progression (5–11), independent of the presence of RF. Consequently, anti-CCP antibody–positive and anti-CCP antibody–negative RA are increasingly viewed as separate disease entities (12). Most studies of the predictive utility of anti-CCP antibodies have investigated either diagnosis or erosive damage, while few have evaluated its utility in clinical practice. In particular, very few studies have compared the value of anti-CCP antibody and RF status in predicting other long-term disease outcomes such as functional disability (7,8), disease activity, and mortality (13).

The clinical utility of anti-CCP antibodies can also be evaluated in terms of response to treatment. Early and aggressive treatment of RA patients is clinically beneficial (14,15). Because anti-CCP antibodies are a marker of disease severity and are detectable early in the disease course, they have the potential to identify those patients with early inflammatory arthritis who will benefit from treatment. Disease severity is predictive of early diagnosis (16) and can trigger the decision to treat. However, disease severity is also predictive of poor treatment response (17); thus, assessment of the differences in responses to treatment by anti-CCP antibody status may be biased due to confounding by indication. Therefore, although anti-CCP antibodies might be a useful marker of who to treat, it is not clear if they will predict those who respond best to treatment.

The aim of this study was 1) to examine the association of anti-CCP antibody and RF status with the long-term outcome of patients with inflammatory polyarthritis (IP), and 2) to examine the differences in response to treatment by anti-CCP antibody status.

## PATIENTS AND METHODS

The patients were recruited from the Norfolk Arthritis Register (NOAR), a primary care–based inception cohort of subjects with recent-onset IP. As described in detail elsewhere (18), the NOAR aims to recruit all adults ages ≥16 years who have swelling of at least 2 joints persisting for at least 4 weeks, and whose symptom onset was after January 1, 1990. The NOAR catchment area covers the former Norwich Health Authority, with notification of cases via general practitioners or hospital attendance. Those who were subsequently diagnosed by a hospital consultant as having a condition other than RA, IP, psoriatic arthritis, or postviral arthritis were excluded. Between 1990 and 1994, 1,098 subjects who satisfied the above criteria were registered with the NOAR. Of these, 913 subjects had a blood sample collected at baseline permitting serologic testing, and thus were included in this analysis.

Written consent was obtained from all of the patients and the study was approved by the Norwich Local Research Ethics Committee.

### Data collection

#### Baseline

Clinical and demographic data (Table 1) were collected by a research nurse via a structured interview and clinical examination shortly after registration (baseline). Detailed information was collected on the use of disease-modifying antirheumatic drugs (DMARDs) and steroids. Each subject completed the Health Assessment Questionnaire (HAQ), modified for use in British patients (19).

**Table 1:** Variables used in the marginal structural weight models*

Variable	Variable type
Demographics	
Age at symptom onset and at each assessment	Decades
Sex	Male
	Female
Months from symptom onset at baseline and at each assessment	Tertiles by assessment
Smoking status at each assessment	Never smoked
	Stopped ≥10 years prior to assessment
	Stopped <10 years prior to assessment
	Current smoker
Serologic and genetic	
Anti-CCP antibody status at baseline (Axis-Shield DIASTAT kit)	<5 units/ml
	≥5 units/ml
CRP level category at baseline and assessment (end-point immunoturbidimetric agglutination)	≤10 mg/liter
	>10 mg/liter
RF status at baseline and assessment (latex agglutination)	<1:40
	≥1:40
Number of copies of the shared epitope	0
	1
	2
Homozygous for the shared epitope	No
	Yes
Disease activity and severity	
DAS28 score at baseline and assessment	
ACR criteria for RA, cross-sectional at baseline and cumulative at assessment	Not met
	Met
Number of swollen, tender, and both swollen and tender joints at baseline and assessment	Tertiles by assessment
Presence of nodules at baseline and assessment	No
	Yes
HAQ score group at baseline and assessment	<1
	≥1 to <2
	≥2
Presence of erosions by assessment	No
	Yes
Larsen score by assessment	Tertiles by assessment
Physical component score of the SF-36	
Mental component score of the SF-36	
Diagnosed with any of 14 defined comorbidities by assessment	No
	Yes
Treatment and hospital attendance	
Attended/referred to hospital since last assessment	No
	Yes
Treated with SSZ, MTX, other DMARDs, and steroids at baseline and by assessment	No
	Yes
Ceased treatment since last assessment or by assessment due to inefficacy or adverse event	No
	Yes
Remission by baseline and assessment	No
	Yes

* Anti-CCP = anti–cyclic citrullinated peptide; CRP = C-reactive protein; RF = rheumatoid factor; DAS28 = Disease Activity Score in 28 joints; ACR = American College of Rheumatology; RA = rheumatoid arthritis; HAQ = Health Assessment Questionnaire; SF-36 = Medical Outcomes Study Short Form 36; SSZ = sulfasalazine; MTX = methotrexate; DMARDs = disease-modifying antirheumatic drugs.

A blood sample was also taken for RF, anti-CCP antibody, and C-reactive protein (CRP) level testing. RF was measured using a latex agglutination technique (positivity: titer of ≥1:40). Anti-CCP antibodies were tested using the Axis-Shield DIASTAT kit according to the manufacturer's instructions (positivity: concentration ≥5 units/ml). CRP levels (mg/liter) were measured using an end-point immunoturbidimetric agglutination method. The Disease Activity Score in 28 joints (DAS28) was calculated using CRP level and 28 tender and 28 swollen joint counts (online at: http://www.das-score.nl/www.das-score.nl/index.html).

#### Followup

Annual assessments were carried out for 3 years, and then at the fifth year. Dates of starting and stopping any DMARD therapy were recorded. A blood sample was taken from all of the subjects at the fifth assessment for RF and CRP level testing. The American College of Rheumatology (ACR; formerly the American Rheumatism Association) 1987 classification criteria for RA (20) were applied both cross-sectionally at baseline and cumulatively at each subsequent assessment (21).

Patients attended for radiographs of their hands and feet at the first and/or second assessments if they had already satisfied the ACR criteria for RA or if the presence of erosions would lead to their satisfying these criteria. All of the patients were invited for radiographs at the 5-year followup. Radiographs were scored using the Larsen method (22) by 2 rheumatologists and major disagreements were arbitrated by a third (23). The patients completed the Medical Outcomes Study Short Form 36 (24), a validated generic health status measure, at the third and/or fifth years (25).

Where relevant, the Office for National Statistics provided details of the patients' deaths, including the cause and date of death. In the analysis, each patient was followed from the date of baseline assessment until death or 5 years from baseline, whichever was sooner.

### Statistical analysis

The patients were placed into 1 of 4 autoantibody groups, determined by serologic assessment of baseline serum: anti-CCP antibody negative/RF negative, anti-CCP antibody negative/RF positive, anti-CCP antibody positive/RF negative, or anti-CCP antibody positive/RF positive. Differences in the patient characteristics at baseline between (i.e., all 4 groups) and within (e.g., anti-CCP antibody–negative subjects by RF status) the autoantibody groups were tested using regression models. Depending on the outcome being tested, the following models were used: median regression for continuous non-normal outcomes, logistic regression for binary outcomes, negative binomial regression for counts, and multinomial logistic regression for categorical outcomes. Furthermore, the differences in patient characteristics at baseline between anti-CCP antibody–positive/–negative patients were tested using the following: the Kruskal-Wallis test for continuous outcomes and the chi-square test for categorical outcomes. The differences in outcomes by 5 years between anti-CCP antibody–positive/–negative patients were investigated (linear regression for changes in continuous outcomes [adjusted by baseline] and logistic regression for the odds of categorical outcomes by 5 years).

### Treatment history

To assess the impact of treatment, particularly early treatment, by anti-CCP antibody status on 5-year outcome, the change in HAQ model was then stratified by treatment history. The time from disease symptom onset to the date of first treatment (any DMARDs/steroids) was categorized as early (<6 months) or late (≥6 months). The duration of any DMARD/steroid treatment, including combination therapies, was stratified into the number of months of treatment in the first 6 months since symptom onset and the number of months of treatment after the first 6 months since symptom onset.

### Marginal structural models

We have previously used a propensity score (26) to adjust for “confounding by indication.” However, propensity methods assume a single treatment decision made at a predetermined point in time, whereas in practice, treatment is likely to change in response to changes in disease severity. These changes in severity may also affect the HAQ score at the end of 5 years, so if we do not take them into account, we will get a biased estimate of the effect of treatment.

However, we cannot use conventional regression adjustment, since the changes in disease severity will be affected by the previous treatments received. If we include intermediate HAQ scores, for example, in a regression model, we will adjust away part of the effect of treatment and again end up with a biased estimate.

The alternative to regression adjustment for confounding is to use weighting (27). If we consider first the simple case of a single treatment decision, we can assign weights to individuals so that the distributions of all confounding variables are the same in the treated and untreated subjects. For example, if the HAQ score tended to be higher in the treated subjects than in the untreated subjects, we could up-weight untreated subjects with high HAQ scores and down-weight untreated subjects with low HAQ scores, until the distribution of HAQ scores in the untreated subjects was the same as that in the treated subjects, and the HAQ score would no longer be a confounder. In practice, we would want to equalize the distributions of a number of variables, and this can be done by including all of the variables in a logistic regression model to predict the probability of treatment: if we weight to balance the probabilities of treatment between treated and untreated subjects, we will balance all of the variables that make up the propensity score, and therefore none of them will confound the association between treatment and outcome. If the predicted probability of treatment for the i^th^ subject is p_i_, then the appropriate weights to use are 1/p_i_ for treated subjects and 1/(1 − p_i_) for untreated subjects; these are referred to as inverse probability of treatment weights (IPTW) (28). To allow for time-varying confounding, we can calculate weights each time that a treatment decision is made. Weighting individuals by the product of these time-specific weights will eliminate confounding by the history of confounders; the resulting model is referred to as a marginal structural model (29–32).

A similar weighting approach (inverse probability of censoring weight [IPCW]) can be used to remove the bias due to loss to followup. Weighting by the product of the IPTW and IPCW produces a population in which both treatment and loss to followup are independent of any of the variables considered potential time-varying confounders, enabling an unbiased estimate of the effect of treatment to be obtained.

Potential confounders are listed in Table 1. At each assessment, we included the values of all of these variables at the current assessment, at baseline (if different from the current assessment), and at the previous assessment (if different from the current assessment and baseline) into a logistic regression model. The outcome in this model was 1 if the subject was receiving treatment at that assessment, and 0 if not. The product of the assessment-specific weights produces an individual's IPTW. The same variables and methodology were used to calculate the IPCW, the only difference being that the outcome was 1 if the subject was censored before the next assessment.

We also had to consider the problem of missing data, since excluding subjects with missing data can lead to biased estimates (33), as well as reducing statistical efficiency. We imputed missing data using switching regression, an iterative multivariable regression technique that retains an element of random variation in the estimates (34). Within Stata, these methods were incorporated within the ice and uvis programs (35). All analyses were undertaken using Stata, version 9.2.

## RESULTS

### Associations between autoantibodies and patient characteristics at baseline assessment

At baseline, 268 (29.3%) of the 916 subjects were anti-CCP antibody positive and 255 (27.8%) were RF positive (Table 2). There were significant differences in the majority of baseline characteristics between the 4 autoantibody groups. However, the differences in disease activity and severity appear to be restricted to anti-CCP antibody status, irrespective of RF status. Anti-CCP antibody–positive patients had the highest DAS28 scores, irrespective of whether they were RF negative or RF positive (difference in DAS28 −0.13; 95% confidence interval [95% CI] −0.59, 0.33). However, significantly more patients satisfied the ACR criteria for RA if they were RF positive and anti-CCP antibody negative than if they were RF negative and anti-CCP antibody negative (difference 23.6%; 95% CI 11.1, 36.2), probably reflecting that RF is part of the ACR criteria for the classification of RA.

**Table 2:** Baseline characteristics of cohort by anti-CCP antibody/RF groups*

	Anti-CCP antibody negative			Anti-CCP antibody positive			
	RF negative (n = 583)	RF positive (n = 65)	Difference in characteristic (95% CI)	RF negative (n = 78)	RF positive (n = 190)	Difference in characteristic (95% CI)	*P* for all groups
Age at symptom onset, years†	51 (39–65)	56 (46–68)	5 (−1.1, 11.1)	5.6 (44–66)	5.7 (49–67)	1 (−5.3, 7.3)	0.009
Women, no. (%)‡	385 (66)	46 (70.8)	4.7 (−7, 16.4)	51 (65.4)	101 (53.2)	−12.2 (−24.9, 0.5)	0.008
Symptom duration at registration, months†	4 (2–10)	4 (1–9)	0 (−4.1, 4.1)	5 (3–12)	4 (2–10)	−1 (−5.1, 3.1)	0.96
HAQ score (n = 910)†	0.63 (0.25–1.25)	0.5 (0.13–1.25)	−0.13 (−0.62, 0.37)	1.13 (0.38–1.63)	0.75 (0.38–1.63)	−0.38 (−0.89, 0.14)	0.15
CRP level, mg/liter (n = 845)†	3 (0–10)	4 (0–17)	1 (−1.2, 3.2)	12 (3–29)	11.5 (5–31)	0 (−2.33, 2.33)	< 0.001
DAS28 score (n = 845)†	3.75 (2.71–4.77)	3.69 (2.52–5.3)	−0.06 (−0.5, 0.37)	4.49 (3.6–5.59)	4.35 (3.37–5.43)	−0.13 (−0.59, 0.33)	< 0.001
Swollen joint count§	6 (2–12)	5 (1–13)	−1 (−5, 3)	9 (4–16)	8 (4–17)	−1 (−5.2, 3.2)	0.003
Tender joint count§	7 (2–16)	7 (2–18)	0 (−4.1, 4.1)	8 (4–17)	8 (4–16)	0 (−4.2, 4.2)	0.98
Swollen and tender joint count§	3 (0–8)	3 (0–10)	0 (−4.1, 4.1)	4 (1–10)	4 (1–9)	0 (−4.1, 4.1)	0.38
Satisfy ACR criteria for RA, no. (%)‡	203 (34.8)	38 (58.5)	23.6 (11.1, 36.2)	44 (56.4)	150 (78.9)	22.5 (10.1, 35)	< 0.001
Smoking status at baseline, no. (%)¶							< 0.001
Never smoked	201 (34.7)	20 (30.8)	−3.9 (−15.8, 7.9)	28 (35.9)	41 (21.7)	−14.2 (−26.4, −2)	
Ex-smoker for ≥10 years	163 (28.2)	14 (21.5)	−6.6 (−17.3, 4)	16 (20.5)	40 (21.2)	0.7 (−10, 11.3)	
Ex-smoker for <10 years	76 (13.1)	14 (21.5)	8.4 (−2, 18.8)	18 (23.1)	37 (19.6)	−3.5 (−14.4, 7.4)	
Current smoker	139 (24)	17 (26.2)	2.1 (−9.1, 13.4)	16 (20.5)	71 (37.6)	17.1 (5.7, 28.4)	

* Values are the median (interquartile range) unless otherwise indicated. Anti-CCP = anti–cyclic citrullinated peptide; RF = rheumatoid factor; 95% CI = 95% confidence interval; HAQ = Health Assessment Questionnaire; CRP = C-reactive protein; DAS28 = Disease Activity Score in 28 joints; ACR = American College of Rheumatology; RA = rheumatoid arthritis.

† Median regression was used to test significance between the groups.

‡ Logistic regression was used to test significance between the groups.

§ Negative binomial regression was used to test significance between the groups.

¶ Multinomial logistic regression was used to test significance between the groups.

The differences in 5-year clinical characteristics were also explained by anti-CCP antibodies rather than RF status (data not shown); therefore, all subsequent analysis focused on anti-CCP antibody status only. Patients who were anti-CCP antibody positive at baseline were more likely to be older, male, satisfy ACR criteria for RA, and have more severe disease, including the presence of erosions, compared with those who were anti-CCP antibody negative at baseline (Table 3). In keeping with previous reports, anti-CCP antibody–positive patients were more likely to have smoked (*P* = 0.001).

**Table 3:** Baseline characteristics of cohort by anti-CCP antibody status*

	All IP patients			IP patients assessed at 5 years		
	Anti-CCP antibody negative (n = 648)	Anti-CCP antibody positive (n = 268)	*P*	Anti-CCP antibody negative (n = 515)	Anti-CCP antibody positive (n = 214)	*P*
Age at symptom onset, years†	52 (39–66)	56 (48–66)	< 0.001	51 (40–64)	55.5 (47–64)	0.002
Women, no. (%)‡	431 (66.5)	152 (56.7)	0.005	356 (69.1)	129 (60.3)	0.023
Symptom duration at registration, months†	4 (2–10)	5 (2–10.5)	0.23	4 (2–10)	5 (2–10)	0.55
HAQ score (n = 910)†	0.625 (0.25–1.25)	0.88 (0.375–1.625)	< 0.001	0.625 (0.25–1.25)	0.875 (0.375–1.625)	0.003
CRP level, mg/liter (n = 845)†	3 (0–11)	12 (4–31)	< 0.001	3 (0–10)	10 (3–23)	< 0.001
DAS28 score (n = 845)†	3.73 (2.66–4.8)	4.4 (3.4–5.45)	< 0.001	3.76 (2.78–4.82)	4.41 (3.43–5.37)	< 0.001
Swollen joint count†	6 (2–12)	8 (4–16.5)	< 0.001	6 (2–12)	8 (4–16)	< 0.001
Tender joint count†	7 (2–16)	8 (4–16)	0.17	7 (2–17)	8 (4–16)	0.29
Swollen and tender joint count†	3 (0–8)	4 (1–9)	0.002	3 (0–8)	4 (1–9)	0.006
Satisfy ACR criteria for RA, no. (%)‡	241 (37.2)	194 (72.4)	< 0.001	203 (39.4)	155 (72.4)	< 0.001
Smoking status at baseline, no. (%)‡			0.001			0.025
Never smoked	221 (34.3)	69 (25.8)		180 (35.2)	60 (28)	
Ex-smoker for ≥10 years	177 (27.5)	56 (21)		141 (27.6)	48 (22.4)	
Ex-smoker for <10 years	90 (14.0)	55 (20.6)		70 (13.7)	42 (19.6)	
Current smoker	156 (24.2)	87 (32.6)		120 (23.5)	64 (29.9)	

* Values are the median (interquartile range) unless otherwise indicated. Anti-CCP = anti–cyclic citrullinated peptide; IP = inflammatory polyarthritis; HAQ = Health Assessment Questionnaire; CRP = C-reactive protein; DAS28 = Disease Activity Score in 28 joints; ACR = American College of Rheumatology; RA = rheumatoid arthritis.

† Kruskal-Wallis test was used to test significance between the groups.

‡ Chi-square test was used to test significance between the groups.

### Differences in treatment over 5 years by anti-CCP antibody status

Patients who were anti-CCP antibody positive at baseline were more likely to have been treated with DMARDs/steroids by 5 years than those without anti-CCP antibodies (85.4% versus 37.5%; *P* < 0.001) (Table 4). Over the 5 years of followup, anti-CCP antibody–positive patients who received treatment were more likely to receive sulfasalazine (SSZ; *P* < 0.001), methotrexate (MTX; *P* < 0.001), or other DMARDs (*P* < 0.001), whereas anti-CCP antibody–negative patients who received treatment were more likely to receive steroids (*P* < 0.001). As the anti-CCP antibody status of the patients was unknown at the time of treatment, these differences reflect anti-CCP antibody status as a marker of disease severity. There were no differences in the time to and on first treatment by anti-CCP antibody status. However, anti-CCP antibody–positive patients were receiving treatment for a longer period of time over the followup (*P* < 0.001).

**Table 4:** Treatment characteristics over 5 years of followup by anti-CCP antibody status*

	Anti-CCP antibody negative (n = 648)	Anti-CCP antibody positive (n = 268)
Treated with DMARDs/steroids over 5 years		
No	405 (62.5)	39 (14.6)
Yes	243 (37.5)	229 (85.4)
Treatment ever over 5 years†		
Sulfasalazine	128 (52.7)	173 (75.5)
Methotrexate	63 (25.9)	110 (48.0)
Steroids	104 (42.8)	83 (36.2)
Other DMARDs	29 (11.9)	33 (14.4)
First treatment type†		
Sulfasalazine	119 (49.0)	161 (70.3)
Methotrexate	32 (13.2)	28 (12.2)
Steroids	90 (37.0)	46 (20.1)
Other DMARDs	20 (8.2)	20 (8.7)
Response to first treatment over 5 years†		
Still on	49 (20.2)	41 (17.9)
Changed combination	136 (56.0)	93 (40.6)
Stopped	47 (19.3)	93 (40.6)
Time to first treatment, median (IQR) months	6 (2–18)	7 (4–15)
Time on first treatment, median (IQR) months	21 (6–54)	20 (5–49)
Time on any treatment over 5 years, median (IQR) months	50.5 (20.5–61)	58 (39–62)

* Values are the number (percentage) unless otherwise indicated. Anti-CCP = anti–cyclic citrullinated peptide; DMARDs = disease-modifying antirheumatic drugs; IQR = interquartile range.

† Percentages are of those treated.

### Associations between anti-CCP antibodies and outcomes by 5 years of followup and differences in treatment response

All 5-year disease outcomes were worse in patients who were anti-CCP antibody positive at baseline both in absolute value and in change from baseline (Table 5). As with baseline characteristics, these differences were independent of RF status (data not shown). Anti-CCP antibody–positive patients experienced a significant increase in HAQ score over the 5 years (mean change 0.20; 95% CI 0.09, 0.31), whereas anti-CCP antibody– negative patients experienced no change in HAQ score (mean 0.005; 95% CI −0.06, 0.07).

**Table 5:** Five-year outcomes by anti-CCP antibody status*

5-year outcome	Anti-CCP antibody negative (n = 515)	Anti-CCP antibody positive (n = 214)	Anti-CCP antibody positive vs. anti-CCP antibody negative (adjusted by baseline)
HAQ score, median (IQR)	0.625 (0–1.375)	1.13 (0.5–1.875)	
Change in HAQ score, mean (95% CI)†	0.005 (−0.06, 0.07)	0.20 (0.09, 0.31)	
Mean difference (95% CI)			0.27 (0.16, 0.39)
Swollen joint count, median (IQR)	0 (0–2)	3 (0–6.5)	
Change in swollen joint count since baseline, mean (95% CI)‡	−7 (−8, −6)	−6 (−7, −4)	
Mean difference (95% CI)			2.79 (1.98, 3.6)
Tender joint count, median (IQR)	1 (0–6)	2 (0–9.5)	
Change in tender joint count since baseline, mean (95% CI)‡	−7 (−8, −5)	−4 (−6, −2)	
Mean difference (95% CI)			2.54 (0.96, 4.12)
Swollen and tender joint count, median (IQR)	0 (0–1)	1 (0–4)	
Change in swollen and tender joint count since baseline, mean (95% CI)‡	−4 (−5, −4)	−3 (−4, −1)	
Mean difference (95% CI)			2.07 (1.35, 2.79)
DAS28 score, median (IQR)	2.22 (1.42–3.09)	3.09 (2.17–4.23)	
Change in DAS28 score, mean (95% CI)§	−1.33 (−1.49, −1.17)	−0.92 (−1.25, −0.6)	
Mean difference (95% CI)			0.78 (0.52, 1.05)
Satisfy ACR criteria cumulatively for RA, no. (%)¶	317 (61.6)	205 (95.8)	
OR (95% CI)			14.23 (7.13, 28.39)
Presence of erosions, no. (%)#	135 (29.4)	162 (80.6)	
OR (95% CI)			9.97 (6.66, 14.92)

* Anti-CCP = anti–cyclic citrullinated peptide; HAQ = Health Assessment Questionnaire; IQR = interquartile range; 95% CI = 95% confidence interval; DAS28 = Disease Activity Score in 28 joints; ACR = American College of Rheumatology; RA = rheumatoid arthritis; OR = odds ratio.

† Linear regression was used to compare outcomes between the groups. The HAQ was completed at baseline and 5 years by 710 subjects.

‡ Linear regression was used to compare outcomes between the groups. Joints were examined at baseline and 5 years on 528 subjects.

§ Linear regression was used to compare outcomes between the groups. The DAS28 score was calculated at baseline and 5 years for 419 subjects.

¶ Logistic regression was used to compare outcomes between the groups.

# Logistic regression was used to compare outcomes between the groups. Radiographs were examined at baseline and 5 years on 660 subjects.

Significant differences in the disease outcomes in anti-CCP antibody–positive/–negative patients remained following adjustment for baseline value, age at onset, and sex (Table 5). Anti-CCP antibody–positive patients had significantly worse outcomes in HAQ scores, joint counts, and DAS28 scores, had higher odds of erosions, and were more likely to satisfy the ACR criteria for RA by 5 years than anti-CCP antibody–negative patients.

Anti-CCP antibody–positive patients benefited less from treatment, particularly early treatment, than anti-CCP antibody–negative patients in terms of functional disability (Table 6). Compared with anti-CCP antibody–negative patients who were never treated, anti-CCP antibody–negative patients who were treated early experienced a significant improvement in functional disability by 5 years, after allowing for treatment history and censoring over the followup (mean adjusted difference in change in HAQ score −0.31; 95% CI −0.53, −0.09). Furthermore, anti-CCP antibody–negative patients treated within the first 6 months experienced a significant improvement in HAQ score compared with those treated later (mean adjusted difference in change −0.52; 95% CI −0.89, −0.14). There was no significant change in HAQ score in anti-CCP antibody–positive patients treated early compared with those anti-CCP antibody–positive patients never treated (−0.14; 95% CI −0.52, 0.24). There was also a nonsignificant improvement in HAQ score in those anti-CCP antibody–positive patients treated within the first 6 months compared with those treated later (−0.25; 95% CI −0.62, 0.11). Anti-CCP antibody–negative patients experienced significant additional benefit for each additional month of early treatment (−0.13; 95% CI −0.22, −0.04), whereas anti-CCP antibody–positive patients did not (−0.05; 95% CI −0.18, 0.07).

**Table 6:** Five-year change in HAQ score comparison of anti-CCP antibody status and treatment*

			Unadjusted		Adjusted by marginal structural model	
	Anti-CCP antibody negative, no.	Anti-CCP antibody positive, no.	Anti-CCP antibody negative	Anti-CCP antibody positive	Anti-CCP antibody negative	Anti-CCP antibody positive
Never treated with DMARDs/steroids over 5 years	305	22	Referent	Referent	Referent	Referent
Treated with DMARDs/steroids over 5 years	185	186	−0.02 (−0.16, 0.11)	0.07 (−0.26, 0.4)	−0.11 (−0.33, 0.11)	−0.02 (−0.31, 0.27)
Time to first DMARD/steroid treatment from symptom onset						
<6 months	88	70	−0.21 (−0.39, −0.03)	−0.06 (−0.42, 0.29)	−0.31 (−0.53, −0.09)	−0.14 (−0.52, 0.24)
≥6 months	97	116	0.15 (−0.02, 0.32)	0.15 (−0.19, 0.49)	0.21 (−0.11, 0.52)	0.11 (−0.18, 0.4)
Effect per number of months of treatment over 5 years						
No. of months treated in first 6 months since symptom onset	–	–	−0.11 (−0.18, −0.04)	−0.05 (−0.13, 0.03)	−0.13 (−0.22, −0.04)	−0.05 (−0.18, 0.07)
No. of months treated after first 6 months since symptom onset	–	–	0.002 (−0.006, 0.01)	−0.005 (−0.01, 0.001)	0.002 (−0.006, 0.01)	0.0006 (−0.008, 0.009)

* Values are the mean difference (95% confidence interval) unless otherwise indicated. HAQ = Health Assessment Questionnaire; anti-CCP = anti–cyclic citrullinated peptide; DMARDs = disease-modifying antirheumatic drugs.

## DISCUSSION

In this primary care–based inception cohort of patients with recent-onset IP, we found an association between the presence of anti-CCP antibodies at baseline and more severe disease both early in the disease and at 5 years of followup, and this association was independent of RF. As a marker of disease severity, anti-CCP antibodies not only predict disease outcomes but also the treatment given over the 5 years. To our knowledge, this is the first observational study to examine the influence of anti-CCP antibody status on treatment response as assessed by change in functional disability. We found that anti-CCP antibody–positive patients with IP showed less benefit from treatment, particularly early treatment, than anti-CCP antibody–negative patients.

Several studies have shown the predictive value of anti-CCP antibodies for radiographic progression (5–11), although the independent role of the antibody in comparison with RF has not yet been confirmed. Some investigators showed that both RF and anti-CCP antibodies are independent predictors (6,7,10,11), whereas others have shown that anti-CCP antibodies rather than RF are the independent predictor of radiographic progression (9), in particular in those patients who were seronegative for RF (8,36). The predictive value of anti-CCP antibodies in seronegative patients was also seen in terms of functional disability (8), although RF rather than anti-CCP antibodies was a predictor of functional disability in a multiple regression model (7). These contradictory results may be a result of studying patients with different durations of disease. The presence of anti-CCP antibodies precedes RF, and indeed, anti-CCP antibodies may be found many years before disease onset (37). In our analysis of patients with early IP, we found that at baseline and 5 years, all of the disease outcome measures were differentiated by anti-CCP antibody status rather than RF at baseline. In particular, in RF-negative patients, the disease outcomes were worse for those with anti-CCP antibodies than those without at baseline. This provides evidence that the marker of disease severity early in the disease process is anti-CCP antibodies rather than RF. Furthermore, the restriction of some studies to patients who satisfy the ACR criteria for RA early in the disease course might have masked the predictive role of anti-CCP antibodies in comparison with RF, as the latter forms part of the criteria for the classification of RA. We have previously shown that anti-CCP antibodies are associated with premature mortality (38). The only other study to compare the association of mortality and anti-CCP antibodies found in a cross-sectional cohort of established RA patients that RF, but not anti-CCP antibodies, was associated with mortality (13). However, high anti-CCP antibody titers were associated with mortality in that cohort.

Further evidence that anti-CCP antibodies are a marker of disease severity is provided by the differences in treatments given over the 5 years by anti-CCP antibody status at baseline. At the time of treatment, the anti-CCP antibody status was unknown and thus cannot have directly influenced treatment decisions. Nevertheless, anti-CCP antibody–positive patients were more likely to be prescribed SSZ, whereas anti-CCP antibody–negative patients were more likely to have been prescribed steroids. In a longitudinal study, Rönnelid et al found no difference in the proportion of patients initially treated with DMARDs by anti-CCP antibody status (39). However, patients who were positive for anti-CCP antibodies were treated longer with SSZ or combination therapy, whereas anti-CCP antibody–negative patients were more often treated with auranofin. In a recent-onset RA cohort, DMARD treatment was more often started in patients positive for anti-CCP antibodies and antimalarials were more likely to be given to anti-CCP antibody–negative patients (40). These differences in treatment given might account for the differences in the response to treatment we have seen. However, after adjusting for disease severity over the followup, we found that those patients negative for anti-CCP antibodies showed more benefit from treatment, particularly early treatment. Furthermore, we did not find a significant difference in the change in HAQ score between those ever treated with MTX and SSZ using the marginal structural model, indicating that this model was successful in accounting for differences in treatments (data not shown).

Because our cohort was recruited and followed in the prebiologics era, the comparison of treatment response is for traditional DMARDs, and we cannot comment on the influence of anti-CCP antibody status in response to biologics. It is unclear if treatment (either traditional DMARDs or anti–tumor necrosis factor [anti-TNF] therapy) alters anti-CCP antibody levels. Some studies have shown that DMARDs (40) and anti-TNF therapies (41–44) reduce anti-CCP antibody titers, whereas others have shown no change in anti-CCP antibody titers but a reduction in RF titers (45–47). Other studies have shown that anti-CCP antibody positivity or titer predicts poor response to anti-TNF treatment (48–51). Our study confirms this association in functional disability, while adjusting for the disease severity marked by anti-CCP antibodies. In a randomized trial comparing 4 targeted treatment strategies, anti-CCP antibody and RF status only predicted progressive disease in the sequential monotherapy group and not the combination therapy groups (52), suggesting that anti-CCP antibodies are not a universal predictor of poor treatment response. Taken together with these previous studies, our work shows that anti-CCP antibody–negative patients do benefit from treatment such as steroids and monotherapy, whereas anti-CCP antibody–positive patients require more aggressive therapies. Furthermore, clinical trials in RA and IP should stratify for anti-CCP antibody status.

This study used a primary care–based cohort of subjects with IP, and therefore had limited selection bias. The strength of studying all IP cases rather than only those subjects classified as RA is that we will have included subjects with milder disease and provided a valuable insight into treatment response in this group. There was a relatively high proportion of patients with neither autoantibody at baseline. It is recognized that patients may seroconvert over the subsequent years, particularly with regard to RF, and thus there may have been some misclassification. However, our study shows that early in the disease course, anti-CCP antibody status predicts disease outcome and treatment response in the long term. Furthermore, the association between features at baseline and anti-CCP antibody rather than RF status was also seen at 5 years. The use of IP subjects could explain why anti-CCP antibody–negative patients have better outcomes, even on adjustment for disease activity, due to a combination of self-limited disease and whether they progress to develop RA. However, this is unlikely to be the case, and restricting the analysis shown in Table 6 to those who met ACR criteria for RA by 5 years gave the same results as using all IP subjects (data not shown).

Adjusting for a series of treatment decisions and fluctuating disease activity over 5 years is complex, and a variety of methods have been suggested, including propensity scores (53), as we have used previously (26,54). Here we have used marginal structural models because they are the only method that adjusts for time-varying confounders. There is a debate as to which variables should be included in the models to estimate the weights (55). Rubin (56) and Robins et al (57) recommend the inclusion of variables even with weak effects on outcome (although strong associations with treatment) in order to minimize bias. Results from simulation experiments (55) suggest that variables related to outcome but not to treatment should always be included because they decrease the variance of estimates without increasing bias, i.e., overfitting is a positive aspect of these models. However, the possibility of residual confounding remains due to unmeasured variables that are associated either with treatment decisions or with outcome. Although we included variables that cover the full spectrum of disease activity, previous treatment, and comorbidity, unmeasured confounders might include patient preferences and compliance. We were able to test (Hosmer-Lemeshow goodness-of-fit test) if the models used to estimate the weights were successful in predicting changes in both treatment and loss to followup, and the IPTW and IPCW models were very successful (*P* = 0.81 and *P* = 0.82, respectively).

Consideration is also required of the number of variables included in the models used to produce the weights and in what form they were included. The recommended sample size is between 200 and 400 subjects or 10–20 cases for each variable included (58,59). Therefore, we had adequate power to use marginal structural models in this context, i.e., 916 subjects with 23 variables. Because the weights produced from the models rather than the estimates directly were used, the variables are included based on how the clinician would use that information in their decision rather than on the efficiency of any estimates.

Does this mean that anti-CCP antibody testing should replace RF in routine clinical practice? From this work, it would appear that their use in conjunction should remain (60). Our study provides further evidence for the reevaluation of the 1987 classification criteria for RA, and support for including anti-CCP antibody status as well as RF status. Anti-CCP antibody–negative patients benefit from monotherapy, whereas anti-CCP antibody–positive patients require more aggressive therapy.

## AUTHOR CONTRIBUTIONS

All authors were involved in drafting the article or revising it critically for important intellectual content, and all authors approved the final version to be published. Dr. Symmons had full access to all of the data in the study and takes responsibility for the integrity of the data and the accuracy of the data analysis. Study conception and design. Farragher, Lunt, Plant, Bunn, Barton, Symmons.

**Study conception and design.** Farragher, Lunt, Plant, Bunn, Barton, Symmons.

**Acquisition of data.** Plant, Bunn, Barton, Symmons.

**Analysis and interpretation of data.** Farragher, Lunt, Symmons.
